# Singular Monstrosity

**Published:** 1868-12

**Authors:** 


### Singular Monstrosity.

Mrs. F. W., a *primipara*, was taken with labor about 2 o'clock A. M., on July 7th, and called to her assistance a midwife living in her immediate neighborhood. The case progressed very well for some time, when the old lady discovered she had a head and foot presentation, *one* foot presenting at the same time with the head. Upon examination she could only find one foot, searching in vain for the other. This alarmed her considerably, and she requested Mr. W. to get assistance as soon as possible. I was summoned in haste to the aid of the old lady. Upon arrival, I found things as above stated, and came to the conclusion, very naturally, that it was a case of twin labor. By the assistance of a lady present, to press against the foot, to prevent its descent, the head readily passed the inferior strait, and a male child was born, perfect in every particular, weighing as I judged, about five pounds. Upon farther examination, but one foot of the other child could be found. The efforts of nature, however, soon brought an end to affairs by relieving the mother of the second; and disclosed to our astonished vision, another child, perfect from the *epigastric* region up. Perfect chest, arms, hands and head; the left abdominal *parietes* perfect, the left thigh, leg and foot perfect. The right abdominal *parietes* wanting, the right thigh, leg and foot wanting; the right cotyloid cavity was filled with a dark gelatinous substance, and the whole of that region presented a dark livid appearance, covered with a membrane. The abdominal viscera, liver, intestines, etc., falling out, the child of course, though born alive, lived only as long as the life supply from the placenta lasted, which did not exceed two minutes. On the right side of the pelvis, and about level with *iliac crest*, was something resembling a small foot, with the plantar surface outward, and detached for about half its length, on the end of which was a single toe. The testes were well formed, the scrotum entire, with the exception of a very small aperture in the right side of the sac, through which the testes on that side could be seen. The penis was entirely wanting, not a vestige of that organ was to be seen. The bones of the pelvis were as fully formed as usual at birth.—*New Orleans Journal of Medicine.*

